# Ten Sessions of 30 Min tDCS over 5 Days to Achieve Remission in Depression: A Randomized Pilot Study

**DOI:** 10.3390/jcm11030782

**Published:** 2022-01-31

**Authors:** Rémi Moirand, Laetitia Imbert, Frédéric Haesebaert, Gabrielle Chesnoy, Benoit Bediou, Emmanuel Poulet, Jérôme Brunelin

**Affiliations:** 1 INSERM, U1028, CNRS, UMR5292, PSYR2 Team, Lyon Neuroscience Research Center, F-69000 Lyon, France; remi.moirand@ch-le-vinatier.fr (R.M.); laetitia.imbert@etu.univ-lyon1.fr (L.I.); frederic.haesebaert@ch-le-vinatier.fr (F.H.); gabrielle.chesnoy@ch-le-vinatier.fr (G.C.); emmanuel.poulet@chu-lyon.fr (E.P.); 2 Université Claude Bernard Lyon 1, Lyon University, F-69100 Villeurbanne, France; 3 Pôle EST, Centre Hospitalier Le Vinatier, F-69500 Bron, France; 4 Faculté de Psychologie et Sciences de l’Education, Campus Biotech, Université de Genève, 9 Chemin des Mines, CH-1211 Geneva, Switzerland; benoit.bediou@unige.ch; 5 Psychiatric Emergency Unit, Hospices Civils de Lyon Centre Hospitalier Universitaire, F-69008 Lyon, France

**Keywords:** tDCS, brain stimulation, depression, DLPFC, MDD

## Abstract

Although transcranial Direct Current stimulation (tDCS) shows promise in the treatment of major depressive episodes, the optimal parameters and population to target remain unclear. We investigated the clinical interest of a 10 session tDCS regimen in patients with mild to severe treatment-resistant depression, in a pilot double-blind, randomized sham-controlled trial. tDCS was delivered over 5 consecutive days (two 30 min sessions per day separated by at least 2 h, 2 mA). The anode and cathode were placed over the left and the right dorsolateral prefrontal cortex, respectively. One month after tDCS, we observed significantly fewer patients who achieved remission (MADRS_10_ < 10) in the sham group (0 out of 18 patients) than in the active group (5 out of 21 patients; *p* = 0.05). However, no significant difference was observed between the groups regarding the mean scores of severity changes throughout the study period. Bifrontal add-on tDCS delivered twice per day over 5 days, in combination with antidepressant medication, can be a safe and suitable approach to achieve remission in patients with mild to severe treatment-resistant major depressive disorder. However, in regards to the pilot nature and limitations of the present study, further studies are needed before any frank conclusions can be made regarding the use of tDCS with the proposed parameters in clinical settings.

## 1. Introduction

Major depressive disorder (MDD) is a severe and frequent psychiatric condition associated with high disturbance in social functioning; in 2017, the WHO ranked major depression as the third cause of the burden of disease worldwide and projected that the disease will rank first by 2030 [1]. It has been observed that despite a large range of available therapeutic strategies, major depression tends to recur in numerous cases and can become resistant in 30% of cases [2].

In cases of treatment-resistant symptoms, noninvasive brain stimulation (NIBS) techniques have been proposed as promising therapeutic strategies, in combination with antidepressants, or as a monotherapy. Amongst them, transcranial Direct Current stimulation (tDCS) raises a particular interest [3,4], mainly because of its low-cost and the little to no evidence for a serious adverse effect associated with its use in humans [5]. tDCS delivers a weak current (1–2 mA) between one anode and one cathode placed over the scalp of the participant. Several tDCS electrode montages and stimulation parameters have been proposed to alleviate symptoms in patients with MDD, but the optimal parameters remain unclear. Recent evidence-based guidelines for the use of tDCS in neurological and psychiatric disorders have concluded on the probable efficacy of anodal tDCS over the left dorsolateral prefrontal cortex (DLPFC), coupled with cathodal tDCS over the right supraorbital region in non-treatment-resistant MDD [4]. However, recent studies with higher statistical power have proposed the use of a bifrontal montage, with the anode over the left DLPFC coupled with the cathode over the right DLPFC (e.g., [6]). These studies reported promising clinical results and led recent guidelines to consider tDCS as definitely effective in depression [7], however, without identifying the optimal parameters to apply (e.g., the electrode montage, the total number of sessions, the number of sessions per day, etc.) and the clinical characteristics of the patients who could benefit from such a therapeutic intervention.

Hence, there is much room to improve tDCS parameters and strengthen its benefits for patients with depression, and especially those with treatment-resistant MDD [8]. Here, we proposed to shorten the time taken to carry out the tDCS sessions, by delivering two daily sessions of tDCS over 5 consecutive days in patients with treatment-resistant MDD. Delivering 10 sessions with two 30 min sessions per day over 5 days [9] instead of delivering the more common protocol with one daily session delivered over 2 weeks, has numerous benefits for clinical settings and decreases the global cost of the intervention. Moreover, although there is no clear evidence in unipolar depression, it has been reported that delivering two daily sessions is a safe intervention and may induce better clinical benefits than the protocol with one single daily session, in patients with vascular depression [10].

## 2. Materials and Methods

### 2.1. Sample 

In a double-blind, sham-controlled study, 41 patients with MDD according to the DSM 5 criteria were assessed for eligibility and randomly assigned to receive either active or sham tDCS. The allocation was provided by the sponsor of the study (CH le Vinatier, randomization by blocks with a block size of 4, no stratification, 1:1 ratio). 

The severity of depressive symptoms was assessed throughout the study period by a blind rater using the 10-item Montgomery and Asberg Depression Rating Scale (MADRS_10_). At inclusion, patients presented with a MADRS_10_ score >20, even under antidepressant medication at an adequate dose for at least 4 weeks. Concomitant (actual and past) clinically relevant psychiatric and neurological diagnosis and personality disorders were exclusion criteria. The level of treatment-resistance was evaluated with the Maudsley score. After the pre-inclusion visit, the severity of symptoms was assessed 3 times throughout the study period by an investigator blind of the treatment condition: at baseline, after the 10 stimulation sessions (post tDCS, i.e., on the 5th day) and one month later (M1). 

All the participants were right-handed and presented with disabling mild to severe depressive symptoms (MADRS_10_ range 20–36). The antidepressant medication was maintained throughout the study period (dose and molecule). Patients were not involved in a psychotherapeutic approach during the study period. All the participants signed a written informed consent after a description of the study. The study was approved by a local ethics committee on 13 April 2016 (CPP Sud Est III 2016-019B; ANSM 2016-A00415-46). The study was a part of a pre-registered study in a public database prior to its completion on 8 June 2016 (clinicaltrials.gov; registration number NCT02793258). Patients were referred to our clinical unit for Treatment Resistant Depression (Ugo Cerletti Unit, CH Le Vinatier, psychiatric hospital) and were included between 2016 and 2021.

**Figure 1:**
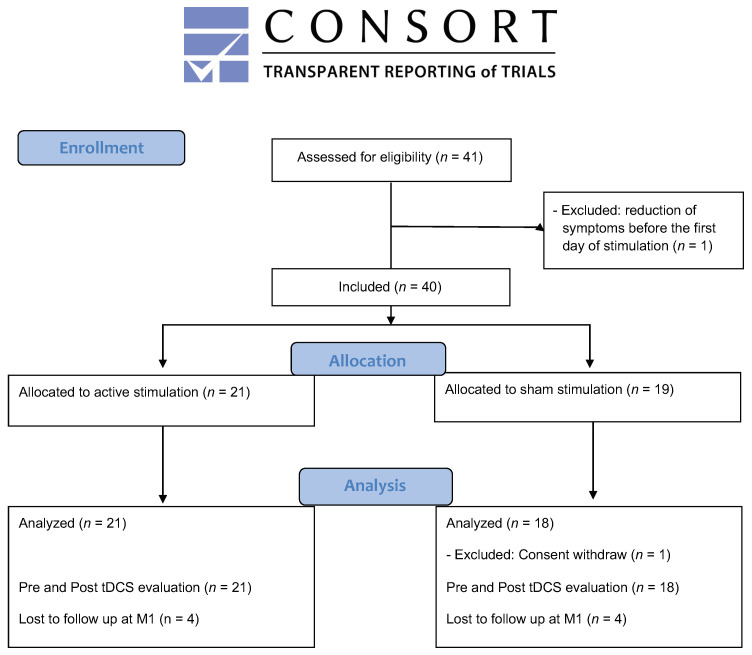
Flow chart diagram of the study (CONSORT 2010).

One patient was not included because he presented with a severe axis II diagnosis (cluster B) that had not been diagnosed during the pre-inclusion visit and reported a drastic reduction in the MADRS score (>80%) between the pre-inclusion visit and the baseline inclusion visit. Another patient, randomly assigned to the sham group, withdrew consent during the stimulation period (no post tDCS data available) and was thus excluded from the analysis. The final analyzed sample consisted of 21 patients in the active group and 18 patients in the sham group (see Figure 1). 

### 2.2. Stimulation Procedure

Stimulation sessions were delivered using a commercial NeuroConn device (Ilmeneau, GmbH). The anode was placed over the left DLPFC (F3 according to the 10/20 EEG international system of electrode placement) and the cathode over the right DLPFC (F4). Electrodes (7 × 5 cm) were placed in saline-soaked sponges with NaCl 0.9%. A tDCS session consisted of delivering 2 mA DC (ramp up/down 30 s) during 30 min. Two sessions per day separated by at least 2 h were delivered over 5 consecutive working days, from Monday to Friday. The first session was generally provided at 11.00 a.m. and the second session at 1.30 p.m. The corresponding total charge per day (calculated as stimulation intensity (A)/electrode size (cm^2^) × duration × number of sessions) was 0.206 C/cm^2^ (total charge per regimen: 1.03 C/cm^2^). The patients were seated in a comfortable chair in a quiet room and they were asked to remain at rest during the stimulation sessions. Sham stimulation was delivered using the commercial “STUDY mode” of the NeuroConn device and consisted of delivering 60 s at 2 mA (ramp up/down 30 s), followed by no stimulation during the remaining 30 min of the stimulation period. Only brief pulses of 110 μA every 550 ms were delivered during this period to control impedance and keep the manipulator blind of the active or sham condition. The blinding was ensured by the use of a unique study code number allocated to each participant and by the device which displayed a continuous measure of impedance during the stimulation sessions. 

### 2.3. Statistical Analysis

The primary outcome of the study was the number of patients who achieved remission at the end point (M1) between the active and sham groups. Remission was defined as a MADRS_10_ score < 10 [11]. Proportions between the groups were compared using Fisher’s Exact test and the odds ratio in JASP (version 0.12.2; Amsterdam, The Netherlands). An additional a posteriori power analysis was performed using G*Power (version 3.1.9.6; Düsseldorf, Germany).

As a secondary outcome, the changes in MADRS_10_ scores throughout the study period (baseline, post tDCS, 1 month follow up) were compared between the 2 groups using a repeated measures ANOVA with 2 factors (GROUP and TIME). The number of responders defined as an at least 50% decrease in MADRS scores was also compared between groups using Fisher’s Exact test. Statistical analyses were undertaken on a strict intention-to-treat sample. The analyses were conducted in a last observation carried forward (LOCF) manner throughout the indicated time points (M1), in case of missing data at M1 (4 patients per group, see Figure 1). 

## 3. Results

No differences were observed between the two groups at inclusion. Further details of the sample characteristics are given in Table 1. The treatment was well tolerated, and no serious adverse event was reported throughout the study period.

### 3.1. Primary Outcome Analysis 

After the 5 days of tDCS (post tDCS), only one patient from the active group was in remission, and none in the sham group. At the end point, one month after the start of the stimulation session, there were significantly (Fisher’s exact *p* = 0.050) fewer patients who achieved remission in the sham group (0/18; 0%) than in the active group (5/21; 24%); corresponding to an odds ratio = 2.512 with 95% confidence interval [−0.458; 5.482]), with a posteriori statistical power (1 − β err prob) = 0.383; actual α = 0.03.

### 3.2. Secondary Outcome Analysis

**Figure 2:**
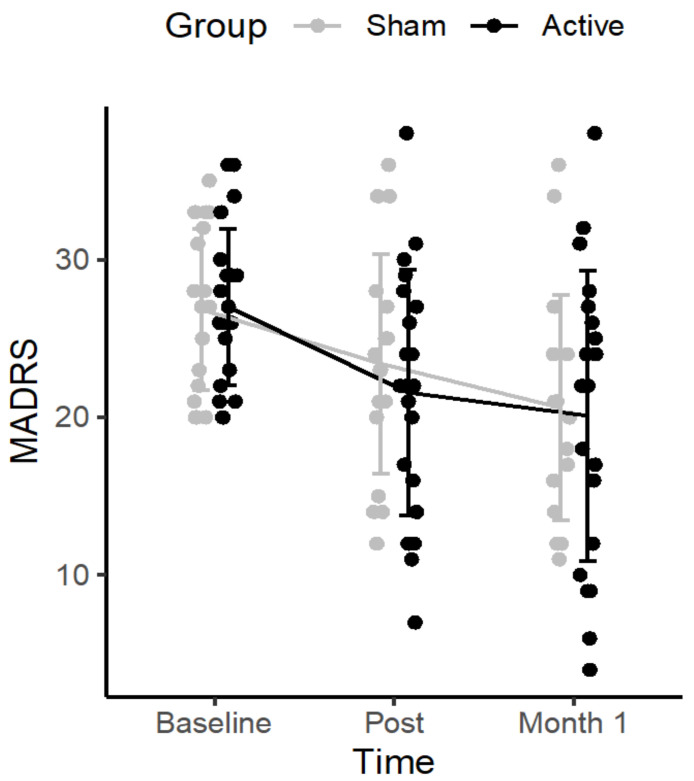
Changes in depressive symptoms severity (MADRS_10_ scores) in patients with major depressive disorders who received 10 sessions of either active (*n* = 21) or sham (*n* = 18) tDCS.

The repeated measures ANOVA revealed a significant effect of TIME (F_(2,74)_ = 17.951, *p* < 0.01, η^2^ = 0.135), but no GROUP*TIME (baseline, day 5, month 1) interaction (F_(2,74)_ = 0.407, *p* = 0.667, η^2^ = 0.003), and no GROUP effect (F_(1,37)_ = 0.150, *p* = 0.701, η^2^ = 0.002). In the active group, the MADRS score decreased from 27.0 (standard deviation (SD): 4.9; range 20–36) to 21.6 (SD: 7.8; range 7–38) post tDCS and to 20.1 (SD: 9.2; range 4–38) at M1. In the sham group, MADRS scores decreased from 26.8 (SD: 5.1; range 20–35) to 23.4 (SD: 7.0; range 12–36) and to 20.6 (SD: 7.1; range 11–36) at M1. A high inter-individual variability was observed (see Figure 2).

No significant difference, but a trend toward significance, was observed between groups regarding the number of responders at the end point (>50% decrease from the baseline), one month after the stimulation sessions (1/18 in the sham group versus 6/21 in the active group; *p* = 0.098); there was only one responder in the active group at day 5 (the one also achieving remission criteria).

## 4. Discussion

The aim of the present study was to investigate the clinical effects of 10 sessions of bifrontal tDCS delivered twice daily over 5 consecutives working days, on the symptoms of patients with MDD. Although active tDCS was not superior to sham intervention to reduce clinical symptoms measured with the MADRS_10_ scores throughout the study period, we observed a significantly higher number of patients achieving remission criteria at the endpoint in the active group (24%) as compared with the sham group (0%). The results are in line with current literature suggesting that active tDCS is superior to sham intervention to achieve remission (odds ratio = 2.12, 95% CI = 1.42, 3.16 [3]). However, we were not able to replicate the findings in depressive symptoms assessed as a mean decrease in MADRS_10_ scores [3]. These findings can be partly explained by the significant sham effect observed in the current study. This sham effect, that was already observed in the tDCS field [12], can reflect either a global effect of the psychiatric care within the protocol with two daily visits, or a biological effect of the sham stimulation itself [13]. This procedure was chosen to maintain the blinding of the participants and tDCS experimenters. Although the sham effect seems significant over time on depressive symptoms, the intensity of the effect did not allow the patients from this group to achieve remission, suggesting the superiority of active tDCS over sham stimulation. 

Although no acute effects of tDCS were observed immediately after the 5 days of stimulation, we observed a superiority of the active over the sham stimulation at the follow up regarding the number of remitters, one month after the simulation sessions. This is in line with other NIBS studies that have reported a delayed clinical effect of tDCS in patients with MDD [14], as well as of repetitive transcranial magnetic stimulation (rTMS) over the left DLPFC, in both patients with MDD [15] and patients with negative schizophrenia [16], suggesting the delayed effect of NIBS-induced neural plasticity when applied over the DLPFC. The present results (see Figure 2) are also in line with current literature supporting a high inter-individual variability regarding the response to antidepressants treatment [17]. Further studies investigating predictive markers of remission are needed to determine patients who will or will not benefit from tDCS treatment, using clinical (e.g., the level of treatment-resistance), demographical [18], or biological markers [19]. This is of major importance to avoid giving false hope in treatment-resistant patients with a long history of depression, and to prevent engaging them in time-consuming protocols.

The present results may also support the clinical interest of delivering two sessions of tDCS per day to achieve remission. This is in line with previous studies with comparable sample sizes, also reporting the beneficial effects of tDCS with two 30 min sessions per day, in a case-series of six patients with depressive disorders [9], six drug free patients with depression during pregnancy [20], and in a sample of 93 elderly participants [10]. The results are also consistent with studies reporting a beneficial effect of two 20 min sessions per day in 31 patients with depression (e.g., [21]), as well as in 30 patients with schizophrenia [22]. However, these findings are not in line with previous results reporting the lack of superiority of active over sham tDCS, while delivering 5 days of two daily 30 min sessions with an anodal F3 coupled with a cathodal right supra-orbital montage [23], in 24 patients with MDD. Regarding differences in the electrode montage and the stimulation parameters across previous studies, the current results could thus also be interesting because the optimal number of sessions per day and the optimal interval between the two consecutive sessions remain uncertain [24] and could be associated with longer or shorter after-effects on brain plasticity, as revealed by the studies that applied tDCS over the motor cortex [25,26]. Hence, delivering ten 30 min sessions (2 sessions per day, separated by at least 2 h) with anodal F3 and cathodal F4 stimulation could be a safe and suitable approach to achieve remission in MDD, the effect being statistically significant one month after the stimulation sessions. If these results are confirmed in future clinical studies with higher sampling, increasing the number of sessions per day could offer several advantages for clinical settings as compared with standard single daily sessions (e.g., [6]). It could allow a decrease in the number of visits, reduce the risk of drop out, diminish the burden of treatment logistics for many patients who cannot take time away to attend daily clinic visits for a long period of time, and thereby lower the cost of the intervention, while at the same time increasing its accessibility for patients, as proposed in the accelerated tDCS protocols (e.g., up to 5 sessions per day, [27]). 

Some limitations should be acknowledged. First, the size of our sample seems limited and despite an observed odds ratio at 2.5 (comparable with those reported in a recent meta-analysis, [3]) and an actual alpha at 0.03 in the main analysis, the results should be taken with caution since the global power of the primary analysis reaches 38%. Based on the observed incidence of remission in each group from the current study (0% vs. 24%), a future study will need to enroll 72 patients (36 per group) to achieve 90% statistical power (with α = 0.05 and β = 0.1). Despite the double-blind design of the current study, we did not systematically evaluate the quality of the blinding for the participants, the clinical raters, nor the tDCS manipulator. For instance, the tDCS manipulator might observe redness under the electrode only in the patients from the active group at the end of the tDCS session. However, in the current study the clinical rater was an independent investigator who was not in contact with the patients during the stimulation sessions, thus limiting this bias. This could, however, be a limitation as recent work suggested that the choice of sham procedure is an important issue and a source of debate in the tDCS field [13] and especially in MDD [12]. Moreover, we cannot rule out that the sham stimulation we delivered (i.e., ramp up/down 30 s, 60 s at 2 mA, and then 110 μA every 550 ms during 29 min) can have a modulatory impact on the brain, leading to clinical improvement in the sham group [13]. Another limitation regards the total number of delivered stimulation sessions. In the current study, we chose to deliver 10 sessions as it was a classic approach in the field at the time the protocol was written. More recent studies have proposed to deliver a higher total number of sessions (e.g., [6]). One may hypothesize that increasing the number of sessions may enhance tDCS response and remission rates and may, in part, explain why we did not observe a significant effect of active over sham tDCS regarding the mean MADRS_10_ score decreases. However, in the meta-analysis developed by Moffa et al., the number of delivered sessions was not significantly associated with a higher response in patients with depression [3]. This was also the case in patients with schizophrenia, as revealed by a meta-analysis that investigated whether delivering more tDCS sessions is better for obtaining an improved clinical outcome [28]. In depression, only higher “tDCS dose” (in C/m^2^) and sessions duration (20 vs. 30 min) seem to be predictors positively associated with tDCS efficacy [29]. In the current study, we used a high dose and 30 min sessions as this seems to be optimal. One may also suggest that increasing the total number of sessions (e.g., delivering 15 sessions [6]), as well as the number of sessions delivered per day (e.g., up to 5 per day [27]) would be a suitable approach to increase the effectiveness of tDCS in patients and to achieve remission in less than one week, as was recently reported using rTMS [30]. 

Importantly, although no difference was observed between the two groups at baseline, the included patients were on different classes of antidepressant medication at several dosages (i.e., SSRIs, SNRIs, TCAs, MAOIs) and in combination with other molecules from different classes (including benzodiazepine and antipsychotics). The current study did not investigate this major point and further studies are needed to investigate the impact of the medication load and of the different classes of molecules on the tDCS-induced clinical effect. 

## 5. Conclusions

Ten 30 min sessions of tDCS delivered over 5 days in combination with current antidepressant medication is a safe and suitable approach to achieve remission in patients with treatment-resistant mild to severe MDD. However, response profiles are highly heterogenous between patients, justifying further studies to determine the optimal parameters and predictive markers of response. Moreover, the statistical power calculated in the present pilot study indicates that we cannot draw any clear conclusion regarding the efficacy of tDCS in clinical settings, and that the use of tDCS with the proposed parameters in treatment-resistant MDD requires further replications. 

## Figures and Tables

**Table 1:** Clinical and sociodemographic characteristics of patients with major depression at inclusion.

	Sham tDCS	Active tDCS	*p*
*n*	18	21	
Age (years)	51.5 (9.7)	48.1 (9.3)	0.272
Education	15.0 (3.1)	13.5 (2.8)	0.129
Sex (F/M)	12/6	12/9	0.542
Episode duration (months)	15.86 (14.7)	21.43 (14.8)	0.138
Illness duration (years)	22.5 (13.7)	21.8 (12.0)	0.878
MADRS_10_	26.8 (5.1)	27.0 (4.9)	0.918
Maudsley	6.5 (3.5)	7.7 (2.5)	0.243
Antidepressant medication			
SSRIs	11	13	1.000
SNRIs	5	8	0.734
TCAs	6	5	0.723
MAOIs	1	1	1.000
Other medication			
BZD	5	4	0.706
Antipsychotics	2	4	0.667

*p*: Student’s *t*-test, except for sex and medication (Fisher’s Exact test). Medication: Selective serotonin reuptake inhibitors (SSRIs); Serotonin-noradrenaline reuptake inhibitors (SNRIs); Tricyclic antidepressants (TCAs); Monoamine oxidase inhibitors (MAOIs), benzodiazepine (BZD).

## Data Availability

The datasets generated and/or analyzed during the current study are available from the corresponding author on reasonable request.
